# Treatment improvement and better patient care: which is the most important one in oral cavity cancer?

**DOI:** 10.1186/s13014-014-0263-x

**Published:** 2014-11-27

**Authors:** Francesca De Felice, Daniela Musio, Valentina Terenzi, Valentino Valentini, Andrea Cassoni, Mario Tombolini, Marco De Vincentiis, Vincenzo Tombolini

**Affiliations:** Department of Radiotherapy, Policlinico Umberto I “Sapienza”, University of Rome, Viale Regina Elena 326, 00161 Rome, Italy; Department of Maxillofacial Surgery, Policlinico Umberto I “Sapienza”, University of Rome, Viale Regina Elena 326, 00161 Rome, Italy; Department Organs of Sense, Policlinico Umberto I “Sapienza”, University of Rome, Viale Regina Elena 326, 00161 Rome, Italy; Spencer-Lorillard Foundation, Viale Regina Elena 262, 00161 Rome, Italy

**Keywords:** Treatment, Oral cavity, Cancer, Head and neck, Radiotherapy, Surgery, Patient care, Quality of life

## Abstract

Due to substantial improvement in diagnosis and treatment of oral cavity cancer, a better understanding of the patient care needs to be revised. We reviewed literature related to OCC and discussed current general management approaches and related long-term radiation toxicities to emphasize the multidisciplinary team involvement. New technical modalities and patient quality of life parameters should be an integral and paramount state in the clinical evaluation to significantly contribute to reduce secondary side effects.

## Introduction

Oral cavity cancer (OCC) accounts for approximately 28% of all head and neck malignancies. In 2014 there will be estimated 11.920 new patients diagnosed in United States and 2.070 deaths [1]. It is associated with tobacco smoking and alcohol abuse. Most cases occur in males, at a rate of 2:1 relative to female, although this trend is dropping, linked to the increase of tobacco use in women [2]. Over the past 20 years, there has been an increment in the incidence of oral cavity cancer in younger non-smokers and non-drinkers. Recent studies emphasize, in these emerging cancer patient populations, the role of Human Papilloma Virus (HPV) infection, especially types 16 – known as a high oncogenic causative agent in cervical cancer [3,4].

The oral cavity consists of various anatomic sites: upper and lower lips, gingiva-buccal sulcus, buccal mucosa, upper and lower gingiva, alveolar ridge, hard palate, floor of mouth and anterior 2/3 of the mobile tongue. These areas have a rich lymphatic drainage and primary regional node dissemination is to levels I to III [5]. Diagnosis and management are established by clinical examination and imaging evaluation – CT and/or MRI from base of skull to clavicle should be routine to assess the loco-regional extent of the primary tumor; CT ad/or PET should be performed to estimate the presence of distant metastasis [6]. Tumors are conventionally staged according to the American Joint Committee on Cancer classification system (Table 1) [7]. Squamous cell carcinomas represent approximately 90% of OCC; although uncommon, various subtypes are described, such as basaloid squamous cell carcinoma, sarcomatoid carcinoma and verrucous squamous cell carcinoma. These histological variants are correlated with differences in prognosis – good prognosis for verrucous carcinoma, only. The remaining 10% is predominantly adenoido-cystic carcinoma [6]. Stage of disease at diagnosis predicts survival rates. The most significant prognostic factor is cervical lymph-nodes status. Patients without lymph-node metastasis had a 5-year cancer specific survival of 94% compared to 51% in patients with clinically positive cervical lymph nodes [8]. HPV-related tumors may assure better prognosis; otherwise definitive conclusion in survival benefit should not be drawn [9].

**Table 1:** **TNM staging system for oral cavity**

**Primary tumor (T)**	
T1	Tumor ≤2 cm in greatest dimension
T2	Tumor >2 cm and ≤4 cm in greatest dimension
T3	Tumor >4 cm in greatest dimension
T4a	Moderately advanced local disease. Tumor invades adjacent structures only (through cortical bone [mandible or maxilla], into deep [extrinsic] muscle of tongue, maxillary sinus, skin of face)
T4b	Very advanced local disease. Tumor invades masticator space, pterygoid plates, or skull base and/or encases internal carotid artery
**Regional Lymph Nodes (N)**	
N1	Metastasis in a single ipsilateral lymph-node, ≤3 cm in greatest dimension
N2a	Metastasis in a single ipsilateral lymph-node, >3 cm and ≤6 cm in greatest dimension
N2b	Metastasis in multiple ipsilateral lymph-nodes, none >6 cm in greatest dimension
N2c	Metastasis in bilateral or controlateral lymph-nodes, none >6 cm in greatest dimension
N3	Metastasis in lymph-nodes >6 cm in greatest dimension
**Distant metastasis (M)**	
M1	Distant metastasis

Due to substantial improvement in diagnosis and treatment of oral cavity cancer, understanding of the patient care needs to be revised. We reviewed literature related to OCC and discussed current general management approaches and related long-term radiation toxicities to emphasize the need for multidisciplinary team involvement. New technical modalities and parameters for patient’s quality life should be integral and paramount in the clinical evaluation to significantly contribute to reduce secondary side effects.

## Review

### General management

Surgery, radiation therapy (RT), chemotherapy or combinations of these therapeutic modalities represent the classical options for managing OCC. The appropriate strategy is based on stage of disease. The current standard recommendation is surgical resection. The choice of a combined modality approach is indicated in advanced disease. Adjuvant radiation therapy is required for stages > T2, ≥ pN2b, positive or close surgical margin, extracapsular nodal spread, and perineural invasion. Adjuvant chemoradiation may be beneficial in high-risk patients, defined as extranodal extension and/or positive surgical margins [10]. Primary definitive radiotherapy is performed in patients with medical contraindication to surgery or in surgical approaches related to significant functional loss [11]. Considering that physiologic functions may be affected, a multidisciplinary team (surgeon, radiotherapist, oncologist, maxillo-facial prosthodontists, pathologist, and radiologist) evaluation should be the standard.

### Overview

In treating OCC, the goals are provided for best functional results and minimal risk of serious complications. Treatment advances are partly responsible for improvement in survival. Therefore a bigger number of survivors run into long-term consequences of cancer treatment. Nowadays managing and preventing sequelae after surgery, radiation therapy with or without chemotherapy, are paramount [12]. Treatment complications depend on specific site and stage of primary tumor, as well as treatment technique.

The extent of surgical procedure depends on the degree of tissue involvement. Tumor should be resected with transoral, transcervical or via mandibulectomy approach, and elective (cN0) or therapeutic (cN+) neck dissection is performed [11]. Peri- and post- treatment complications include bleeding, infection, fistulas, aspiration and swallowing or speech deficits.

Regarding RT, the risk of radiation-induced toxicities is significant in OCC, considering the anatomic proximity to various structures. The ionizing radiation produces damage in normal tissues located in the treatment field. Improvement in modern RT techniques, such as intensity modulated RT (IMRT), has minimized the severity of late toxicities by reducing dose to organs at risk (OARs). The maximum dose limits of standard fractionation for OARs are listed in Table 2. However, clinical trials are limited by size and there is no clear evidence to support interventions to improve the loss of functional aspects in OCC patients treated with radiation therapy [13]. Thus we will focus on this aspect.

**Table 2:** **Constraint of head and neck organs at risk**

**OAR**	**Theorical constraint**
*Spinal cord*	D max <45 Gy (0.5 cc =48 Gy)
*TMJ*	
Homolateral	D5% ≤70 Gy
Controlateral	D5% ≤70 Gy
*Mandible*	< 65 Gy
*Tooth*	< 60 Gy
*Parotid gland*	
Homolateral	-
Controlateral	D mean ≤26 Gy; D50% ≤30 Gy
*Submandibular gland*	
Homolateral	-
Controlateral	D mean ≤26 Gy; D50% ≤30 Gy

OAR = organ at risk; TMJ = temporomandibular joint; D = dose.

### Patient care: smoke and alcohol, nutritional evaluation and dental evaluation

The severity of radiation therapy-related toxicities imposes an adequate assessment, support and surveillance before, during and after treatment. Multidisciplinary evaluation, according to a uniform policy, is an integral component of patient care. Patient should be educated about preventive strategies. Collaborative efforts between clinicians and patients could potentially sustain changes in habits’ conduct.

Patients should be incited to change their “unnecessary” habits, because smoke and alcohol consumption may reduce the efficacy of treatment and increase the risk of new OCC following the first primary cancer [14]. This etiological background is an important factor in field cancerization [15]. Due to prolonged carcinogen exposure, patients are susceptible for developing a second primary tumor, with a risk 2.8 times greater than expected [16]. Programs that support quitting or limiting consumption of tobacco and alcohol could be beneficial.

Adequate nutritional support is extremely important in OCC patients, to attenuate weight loss. Losing >10% of body weight during therapy is correlated with increased complications and deterioration in global quality of life [17]. Patient should be well-informed about the importance of maintenance of good nutrition. Modification of the consistency of food may be inevitable. Dietary management and supplement drinks could be useful to maintain caloric need; tube placement should be recommended in patients with severe pre-treatment weight loss. Several trials have demonstrated the advantage of prophylactic gastrostomy tube placement to minimize weight loss, in patients submitted to chemoradiotherapy [18].

Before starting radiotherapy a careful and complete dental and oral evaluation is recommended. Extraction of teeth in poor condition should be carried out, to reduce the subsequent risk of oral cavity damage. Due to radiation-induced xerostomia and salivary gland dysfunction, patients are at risk of dental complications, such as caries, dento-alveolar infection, bone demineralization and osteoradionecrosis. An excellent oral hygiene is important and patient should be advised to practice it. Soft toothbrush is available for patient to facilitate daily oral hygiene procedures. Topical fluoride applications should be started and continued after treatment to optimize dental care.

### Sequelae of treatment: focus on xerostomia, trismus, radiation caries and osteoradionecrosis

The late toxicities of radiation therapy protocols – with or without concomitant chemotherapy – included xerostomia, trismus, fibrosis and muscle atrophy, caries, and osteoradionecrosis.

Xerostomia represents the most common side effect of radiation therapy. It is defined as objective – unstimulated saliva flow rate <0.1 ml/min – and subjective – more than 45% reduction in unstimulated saliva flow rate – sensation of dry mouth [19]. Salivary glands have an high impact on quality of life. Saliva plays a central role in dental and oral care, in lubricating mucosa, in contributing to antimicrobial activity. Saliva daily volume is produced by submandibular glands (about 70%; serous and mucous secretion), parotid gland (about 20%; serous secretion) and minor salivary glands (less than 10%; mucous secretion, except Von Ebner serous glands) [20]. The submandibular and the minor salivary glands guarantee oral moinsture; parotid glands provide watery saliva during eating [21]. Details of salivary glands’ characteristic are shown in Table 3 [20].

**Table 3:** **Salivary glands**

**Localisation**	**Gland**	**Type**
Lips	Upper lip	Serous
	Lower lip	Serous
Cheek	Buccal mucosa	Mixed (predominantly mucous)
	*Parotid*	Serous
Hard palate	Postero-lateral hard palate	Mucous
Tongue	Von Ebner (dorsal)	Serous
	Weber Blandin Nuhn (marginal)	Mixed (predominantly mucous)
Floor of mouth	*Submandibular*	Mixed (predominantly serous)
	*Sublingual*	Mixed (predominantly mucous)

*Italic type* indicate major salivary glands.

Damage to major and minor salivary glands causes alteration in quantity, quality and consistency of saliva. Salivary glands are radio-sensitive organs. Hyposalivation depends on reduced water and protein rich secretion in parotid glands and loss of function depends on mucous secretion of submandibular glands and minor salivary glands. Saliva turns into a white and viscous fluid, secondary to pH acidification, decrease in bicarbonate concentration and increase in sodium, calcium and magnesium. In addition to pH and electrolyte levels modifications, immunoproteins concentration is also altered, resulting in deleterious effects on oral flora with increase in Streptococcus mutans, Candida species and Lactobacillus species [22].

Although irradiation-induced damage in salivary gland was first described in 1911, its mechanism is still a debate [23]. Radiation-effect on salivary glands has been tested principally in parotid glands. Regional differences in radio-sensitivity have been, described in rat parotid gland, considered as plausible model for human circumstances [24]. The cranial part of the parotid gland has higher sensitivity to radiation exposure than the caudal part. The mechanism of damage is related to the radiation effects on muscarinic receptor induced secretary responsiveness, with consequent destruction of serous cells and loss of serous properties in saliva [25]. This is a possible explanation for the high radio-sensitivity of the parotid glands compared with the relative radio-resistence of the other major salivary glands, which contain much higher level of mucous cells [26]. According to radiobiological principles, metal ions are the agents responsible for irradiation damage, inducing lipid peroxidation. Therefore the radiosensitivity of salivary glands is linked to their content of secretory granules [27]. The time kinetics of damage is expressed in two phases: at first (0–60 days) salivary gland dysfunction depends on plasma membrane damage, later (60–240 days) on “traditional” killing of progenitor cells [28]. Salivary gland hypofunction and consequent xerostomia can persist and develop more than 5 years after radiation treatment, significantly increasing the risk of oral and dental disease and aggravating the difficulties with speaking and eating [29].

Last decades are characterized by improvement in radiation therapy technology and this guarantees higher rates of salivary glands sparing. IMRT, compared with conventional 3-D radiotherapy, significantly reduces the incidence of xerostomia (at 12 months: 38% vs 74%; at 24 months: 29% vs 83%) and results in better recovery of saliva secretions [30]. However these results are not completely satisfactory, because the analysis had exclusively included oropharynx and hypopharynx cancers and therefore salivary glands are not always included in the target volume. But instead in OCC, due to primary tumor localization, it is necessary to deliver a high dose of radiation to the oral cavity. Consequently, it is much more complicated to protect salivary glands, because of their anatomical location, size and bilateral symmetry, and often glands are in (or close to) the target.

Parotid sparing RT is recommended method for the prevention of xerostomia. The protective effect of Amifostine and Pilocarpine is not so definite. Amifostine administration reduced acute (from 78% to 51%, p-value <0.0001) and chronic (from 57% to 34%; p-value 0.002) xerostomia, but it is also combined with nausea, vomiting and hypotension [31]. Administration of oral Pilocarpine during radiation therapy did not improve xerostomia, whereas its efficacy could be dependent on mean dose to parotid gland [32].

Artificial saliva could ameliorate hyposalivation, but its effect is transient and, considering the cost, most patients prefer the frequent use of water. Stem cell replacement could be a potential valid therapy for xerostomia; further researches are necessary [33].

Trismus is a debilitating condition, defined as reduced jaw mobility, limited to a maximum inter-incisor opening measurements of 35 mm or less. Historically the word trismus was used to indicate a difficulty in opening the mouth, secondary to tetanus spasm of the masticator muscles [34]; nowadays it refers to all conditions of limited mouth opening, such as direct trauma, oral infection, oral surgical procedures, drug history and head and neck tumors [35].

Trismus may be induced by radiation therapy and it is a well known late complication of treatment. According to the Common Terminology Criteria for Adverse Events (CTCAE) v3.0 the degree of trismus is evaluated assessing masticatory dysfunction. It is graded as G1 (decreases range of motion without impaired eating), G2 (decreases range of motion requiring small bites, soft foods or purees) and G3 (decreases range of motion with inability to adequately aliment or hydrate orally). The loss of jaw mobility is generally the result of radiation-induced fibrosis of temporomandibular joint and/or masticatory muscles. Fibrosis process can be divided in three histopathological phases: an initial pre-fibrotic non-specific inflammatory phase, with loss of natural endothelial barrier and direct exposure of connective tissue; a constitutive organized phase, characterized by high density of fibrotic cellular elements; and a late fibroatrophic phase, with tissue densification and remodeling [36]. Trismus is a rapid process during the first 9 months after radiotherapy, and then its progress becomes slower over later months [37]. Trismus occurs with variable incidence and severity, ranging from 5% to 42%, if temporomandibular joint and/or masticatory muscles are included in the target volume [38,39]. High radiation dosage >55 Gy to these structures are related to significantly high incidence of trismus, with an increased risk of 24% with every additional 10 Gy [38,40]. Pterygoid muscles irradiation is responsible for reduction in lateral and protrusive jaw movements. High radiation dose to these muscles correlates to increase in mandibular dysfunction. Goldstein et al. [41] investigated the effects of radiation therapy on jaw opening and they found that irradiation of pterygoid muscles was sufficient to induce trismus in 31% of patients. Recently, different trials showed a better trismus profile in patients receiving IMRT than those receiving 3-D RT, sparing pterygoid muscles, especially lateral pterygoid ones [38,42-44].

The consequences of trismus can be serious; it can cause difficulty in eating, swallowing and maintaining adequate oral hygiene. Appropriate post-radiation trismus therapy is still unknown. Encouraging patient to maintain muscles mobilization is paramount. Specific active and passive exercise, using a TheraBite device or tongue blades, may improve organ’s function, minimizing the effects of radiation [37,45]. Botulinum toxin, injected transcutaneously into the masseter muscle, did not improve trismus [46]. If conservative treatments fail, coronoidectomy should be considered [47].

Radiation caries represent a multifactorial and complex oral cavity complication. Indirect effects are more important than direct radiogenic damage to the dentition [48]. Dentition integrity is influenced by tooth-level radiation dose, reduced salivary flow rate, topical fluoride use, oral hygiene status and time after the end of radiation therapy [49]. Induced caries showed same morphological patterns of decay with natural caries: accelerated demineralization and reduced remineralisation of tooth structure, changes in translucency, reactionary dentin and intralobular dentin deposition [48]. Cariogenesis is predominantly associated with hyposalivation and its related consequences, particularly altered composition of saliva and shift in oral microflora to a highly cariogenic bacteria flora [50]. Radiation-related caries are, therefore, indirect complications of dentition, with a rapid onset and progression [51]. Clinically radiation caries are not related to severe pain. Alteration starts mainly on the labial surface (the tooth surface adjacent to the lips); due to low pH saliva and loss of remineralisation capacity, the minerals of enamel and dentin are easily dissolved; then carious lesion progresses to affect the entire crown and results in increased friability and breakdown of tooth [52]. Instead, direct effects of radiation on tooth structures are still not well-known. Several studies *in vitro* have demonstrated changes in enamel and dentin proprieties at tooth dose greater than 60 Gy [49,53,54]. Although further investigations are necessary to evaluate the real impact of radiation dose on dentition, if possible, in daily practice, a tooth dose <60 Gy should be respected.

Clinical management of radiation-related caries is essentially based on clinical experience and preventive oral health care programs should be paramount. From a dental point of view, maintenance of meticulous oral hygiene, daily use of topical fluoride and control of cariogenic microflora play a central role in radiation caries prevention.

Osteoradionecrosis (ORN) is a well documented late complication of radiation therapy for OCC. It is a slow process, not apt to heal spontaneously, characterized by chronic, painful necrosis associated with late sequestration and permanent bone deformity [55]. Biologically, ORN is characterized by hypoxic, hypocellular and hypovascular tissue, followed by tissue breakdown [56]. Hypoxia and hypocellularity are secondary to radiation-induced activation and dysregulation of fibroblastic activity that caused vascular fibrosis and thrombosis [57]. Because of its relatively poor vascularization, the mandible, especially the buccal cortex of premolar, molar and retromolar regions, is much more vulnerable than other bones of the head and neck district. Mandible is exclusively supplied by the inferior alveolar artery (IAA), a branch of the maxillary artery; therefore the obliteration of the IAA causes an ischemic necrosis in irradiated atrophic tissue [58,59]. Since the first ORN description in 1922, several scales have been proposed to provide a universal scoring system to classify ORN [60-64]. There is, however, a general consensus about the CTCAE v3.0 as grading system for the late effects of radiation. It grades ORN considering clinical presentation, radiographic evidence and medical treatment. ORN is defined as G1 (asymptomatic, radiographic findings only), G2 (symptomatic and interfering with function, but not interfering with activities of daily living; minimal bone removal indicated, such as minor sequestrectomy), G3 (symptomatic and interfering with activities of daily living; operative intervention or hyperbaric oxygen indicated) or G4 (disabling).

Radiation and its characteristics (total dose, fractionation, type of energy, field size) are the principal related factors of ORN. After radiotherapy, patients are at greater risk of ORN. Conditions that predispose are: high radiation dose to mandible >65 Gy, hyperfractionation radiation therapy scheme, volume of the mandible included in the target, and maximal dose coverage close to the bone, such as implant source. Early presentation – less than 2 years after radiation therapy – is correlated to a high radiation dose, whereas late stage – several years after radiation therapy – is associated to trauma within the compromised tissue. The incidence of ORN reported in literature varies from 35% to 0.9%, as a result of differences in the observation periods. The range is most commonly from 11.8% to 3% in recent reports, because of technical progress in radiation techniques [65]. IMRT provides a better target volume dose coverage, sparing OARs and therefore minimizing toxicities [66]. Tooth extraction and implant surgery represent promoting factors in the development of ORN. Traditional thinking about pre-irradiation healthy dental extractions do not appear to reduce the risk of ORN; the only teeth that really need to be extracted before radiation therapy are those in poor condition that will reside in the high dose radiation field [67]. Although Starcke et al. [68] indicated that a shorter time period is not associated with increased ORN risk, an interval period of at least 14 days between extractions and radiation therapy is recommended to allow complete healing of the extraction site. Implant placement well correlates with ORN risk. To reduce the probability of ORN onset, implant surgery should be performed minimum 14 days before radiotherapy or from 6 to 24 month after treatment [69].

The better treatment of ORN is prevention; dental care and oral hygiene represents the key. When ORN occurs, treatment ranges from conservative methods to segmental mandibular resection with free vascularised bone grafting, for advanced stage. However elective surgical procedure must be considered very carefully after irradiation. Based on radiation-induced fibroatrophic damage, new therapeutic regimens have been tested. Pentoxifylline combined with Tocopherol act as antifibrotic agents, increasing bone formation and mucosal healing [70]. The real efficacy of hyperbaric oxygen therapy remains remarkable debate. The rationale is its potential role in stimulating monocyte and fibroblast function and neoangiogenesis. But final results are not satisfactory, thus treatment with hyperbaric oxygen should not be recommended [71].

## Conclusion

OCC remains a great challenge of the head and neck oncological scenario, in achieving local tumor control and overall survival benefit within tolerable long-term toxicity. The main purpose of this article was to provide a rationale and stronger knowledge of issues and to propose methods of prevention of radiation morbidity. Treatment improvement and patient care should advance together.

## Abbreviations

OCC: Oral cavity cancer
HPV: Human Papilloma Virus
RT: Radiation therapy
IMRT: Intensity modulated RT
OARs: Organs at risk
CTCAE: Common Terminology Criteria for Adverse Events
ORN: Osteoradionecrosis
IAA: Inferior alveolar artery
